# *Bacillus velezensis* A2 Can Protect against Damage to IPEC-J2 Cells Induced by Zearalenone via the Wnt/FRZB/β-Catenin Signaling Pathway

**DOI:** 10.3390/toxins16010044

**Published:** 2024-01-13

**Authors:** Jing Cai, Xuanshuai Yuan, Yuhang Sun, Jia Chen, Peng Li, Shuhua Yang, Miao Long

**Affiliations:** Key Laboratory of Zoonosis of Liaoning Province, College of Animal Science and Veterinary Medicine, Shenyang Agricultural University, Shenyang 110866, China; 2018200143@stu.syau.edu.cn (J.C.); 2023240833@stu.syau.edu.cn (X.Y.); syh2019@syau.edu.cn (Y.S.); 2020200157@stu.syau.edu.cn (J.C.); lipeng2018@syau.edu.cn (P.L.); yangshuhua0001@syau.edu.cn (S.Y.)

**Keywords:** Zearalenone, *Bacillus velezensi*s A2, IPEC-J2, Wnt/FRZB/β-catenin

## Abstract

Zearalenone (ZEA) has adverse effects on human and animal health, and finding effective strategies to combat its toxicity is essential. The probiotic *Bacillus velezensis* A2 shows various beneficial physiological functions, including the potential to combat fungal toxins. However, the detailed mechanism by which the *Bacillus velezensis* A2 strain achieves this protective effect is not yet fully revealed. This experiment was based on transcriptome data to study the protective mechanism of *Bacillus velezensis* A2 against ZEA-induced damage to IPEC-J2 cells. The experiment was divided into CON, A2, ZEA, and A2+ZEA groups. This research used an oxidation kit to measure oxidative damage indicators, the terminal deoxynucleotidyl transferase-mediated nick end labeling (TUNEL) method to detect cell apoptosis, flow cytometry to determine the cell cycle, and transcriptome sequencing to screen and identify differentially expressed genes. In addition, gene ontology (GO) and the Kyoto Encyclopedia of Genes and Genomes (KEGG) were adopted to screen out relevant signaling pathways. Finally, to determine whether A2 can alleviate the damage caused by ZEA to cells, the genes and proteins involved in inflammation, cell apoptosis, cell cycles, and related pathways were validated using a quantitative reverse transcription polymerase chain reaction (qRT-PCR) and Western blot methods. Compared with the CON group, the levels of reactive oxygen species (ROS) and malondialdehyde (MDA) in the ZEA group increased significantly (*p* < 0.01), while the levels of antioxidant enzyme activity, total superoxide dismutase (T-SOD), glutathione peroxidase (GSH-PX), total antioxidant capacity (T-AOC), and catalase (CAT) decreased significantly (*p* < 0.01). Compared with the ZEA group, the A2+ZEA group showed a significant decrease in ROS and MDA levels (*p* < 0.01), while the levels of T-SOD, GSH-PX, T-AOC, and CAT increased significantly (*p* < 0.01). TUNEL and cell cycle results indicated that compared with the ZEA group, the A2+ZEA group demonstrated a significant decrease in the cell apoptosis rate (*p* < 0.01), and the cell cycle was restored. Combining transcriptome data, qRT-PCR, and Western blot, the results showed that compared with the CON group, the mRNA and protein expression levels of Wnt10 and β-catenin increased significantly (*p* < 0.01), while the expression level of FRZB decreased significantly (*p* < 0.01); compared with the ZEA group, the expression levels of these mRNA and proteins were reversed. *Bacillus velezensis* A2 can increase the antioxidant level, reduce inflammatory damage, decrease cell apoptosis, and correct the cell cycle when that damage is being caused by ZEA. The protective mechanism may be related to the regulation of the Wnt/FRZB cell/β-catenin signaling pathway.

## 1. Introduction

Zearalenone (ZEA) is an estrogenic fungal toxin produced by *Fusarium oxysporum*, which is ubiquitous and mainly present in moldy grain crops and grain products. ZEA not only affects the safety of feed and food, but also can accumulate in human or animal bodies through the food chain, thereby endangering the health of humans and animals. Research has shown that ZEA poses a significant threat to the health of livestock and poultry, with strong reproductive toxicity [1] and immunotoxicity. ZEA induces excessive ROS generation in organisms [2,3] and can trigger severe oxidative damage in intestinal epithelial cells [4]. Cellular oxidative stress is one of the fundamental causes of damage to biomolecules such as DNA, proteins, and lipids [5]. Research has confirmed that oxidative damage is one of the main toxic mechanisms of ZEA. ZEA increases the cell activity and production of reactive oxygen species (ROS) and malondialdehyde (MDA) in a concentration and time-dependent manner [6,7]. Therefore, if ZEA can be inhibited from causing oxidative damage to intestinal epithelial cells, its toxic effect on internal organs can be reduced to the benefit of both humans and animals.

ROS are closely related to intestinal injury. High concentrations of ROS can attack the sugars, lipids, proteins, and nucleic acids of intestinal endothelial and epithelial cells, leading to oxidative damage to intestinal cells and affecting the structure and function of the intestine [8,9]. Previous research has shown that high doses or long-term exposure to fungal toxins can cause damage to intestinal health [10]. Mycotoxins such as aflatoxin B1, ochratoxin A, vomiting toxin, and ZEA can all lead to intestinal oxidative stress [11]. The results of in vitro experiments indicate that when intestinal epithelial cells are exposed to ZEA, even at low doses, it can exert harmful effects on their survival rate. ZEA can decrease the survival rates of small intestinal porcine epithelial cell lines IPEC-1 and IPEC-J2 [12], and ZEA can induce mitochondrial damage by reducing the activity of antioxidant enzymes, the accumulation of ROS, and reducing mitochondrial membrane potential in IPEC-J2 cells, thereby affecting cell survival [4]. The results of in vivo experiments indicate that adding 0.5 to 1.5 mg/kg of ZEA to feed can cause oxidative damage to the jejunum of weaned piglets, and the Keap1-Nrf2 signaling pathway plays an important protective role in suppressing the oxidative damage process of ZEA to the intestine [13]. ZEA can also induce inflammatory reactions in the intestine, which activate ROS-mediated NLRP3 inflammasomes, thereby promoting the inflammatory cytokine IL-1 β and IL-18 relies on caspase-1 release [14]. The inflammatory mediators in the inflammatory process, such as adhesion factors and interleukins, can be induced by oxidative stress [15]. Therefore, if the damage caused by ZEA to the intestine can be suppressed, it will be beneficial in reducing the toxic effect of ZEA on the body.

Probiotic Bacillus can improve the antioxidant capacity of the intestine. Research has found that *Bacillus coagulans* can enhance the activity of intestinal SOD and CAT, reduce the contents of MDA and H_2_O_2_, and alleviate oxidative damage in the intestinal tract of piglets [16]; *Bacillus coagulans* can alleviate the morphological damage and intestinal inflammation caused by *Salmonella enteritidis* infection [17]. Furthermore, adding *Bacillus subtilis* to feed can enhance the antioxidant capacity of the small intestine and mitochondria [15]. *Bacillus subtilis* cw14 confers a protective effect against the Caco-2 cell barrier damage and cell damage induced by ochratoxin A [18]. Some probiotics such as Lactobacillus and Bacillus can adsorb or degrade ZEA, and also have strong antioxidant functions. Adding probiotics (such as yeast, lactobacillus, and Bacillus) that can degrade or adsorb ZEA in feed can weaken the toxic effects of ZEA on animals by reducing the concentration thereof [19,20,21] (Krifaton et al., 2013; Fu et al., 2018). Cell experiments showed that some probiotics are capable of directly reducing the oxidative damage to cells caused by external toxins [22,23].

This experiment established in vitro models with CON, A2, ZEA, and A2+ZEA groups by taking IPEC-J2 cells as the research object. The detection of the cell cycle by flow cytometry, the measurement of cell apoptosis in each group through TUNEL, and the detection of the expression of inflammatory factors in each group of cells were conducted. Transcriptomics techniques were adopted to compare the transcriptome differences among different groups of cells, and immunoblotting and fluorescence quantitative PCR techniques confirmed the differential expression of related gene proteins. This finding clarified the relevant signaling pathways of A2 intervention in ZEA-induced damage to IPEC-J2 cells, which revealed the toxic process associated with ZEA, further revealing the mechanism of A2 intervention in ZEA-induced damage to porcine epithelial cells. The research results offer a deep understanding of the molecular mechanism of ZEA on intestinal damage in piglets, and also provide a theoretical and practical basis for the use of *Bacillus velezensis* A2 to prevent and control ZEA poisoning.

## 2. Results

### 2.1. Detection Results of IPEC-J2 Cell Oxidation Indicator

**Figure 1:**
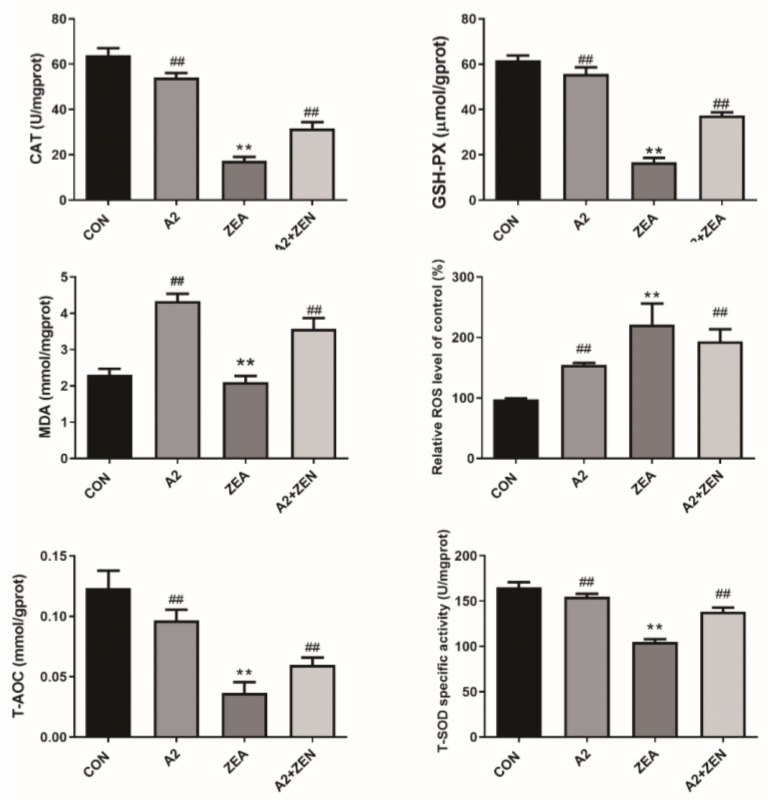
Effects of ZEA and A2 on intracellular oxidation indicators in IPEC-J2 cells. Note: Each group of data has an average of six independent replicates ± SD (*n* = 6), with “**” indicating a significant difference compared to the ZEA and CON groups (*p* < 0.01), and “##” denoting a significant difference between the A2 group or A2+ZEA group and the ZEA group (*p* < 0.01).

As shown in Figure 1, compared with the CON group, the levels of ROS and MDA in the ZEA group significantly increased (*p* < 0.01), indicating that ZEA significantly increased the intracellular oxidation levels. The levels of T-SOD, GSH-PX, T-AOC, and CAT significantly decreased in the ZEA group (*p* < 0.01), showing that ZEA weakened the antioxidant capacity of cells. These results implied that ZEA disrupted the intracellular redox balance, leading to a significant increase in oxidative stress levels and significant damage to the antioxidant defense system. However, compared with the control group, the levels of ROS and MDA were significantly reduced in the A2+ZEA group (*p* < 0.01), indicating that the combination of A2 treatment had a significant inhibitory effect on intracellular oxidation levels. Meanwhile, the levels of T-SOD, GSH-PX, T-AOC, and CAT significantly increased in the A2+ZEA groups (*p* < 0.01), indicating that the combined treatment of A2 improved the antioxidant defense ability of cells. In summary, A2 alleviated cellular oxidative damage and significantly reduced the toxic damage caused by ZEA to IPEC-J2 cells. This outcome indicates that under the oxidative stress caused by ZEA, the application of A2 can significantly enhance the antioxidant capacity of cells and reduce oxidative damage.

### 2.2. Detection Results of IPEC-J2 Cell Apoptosis

**Figure 2:**
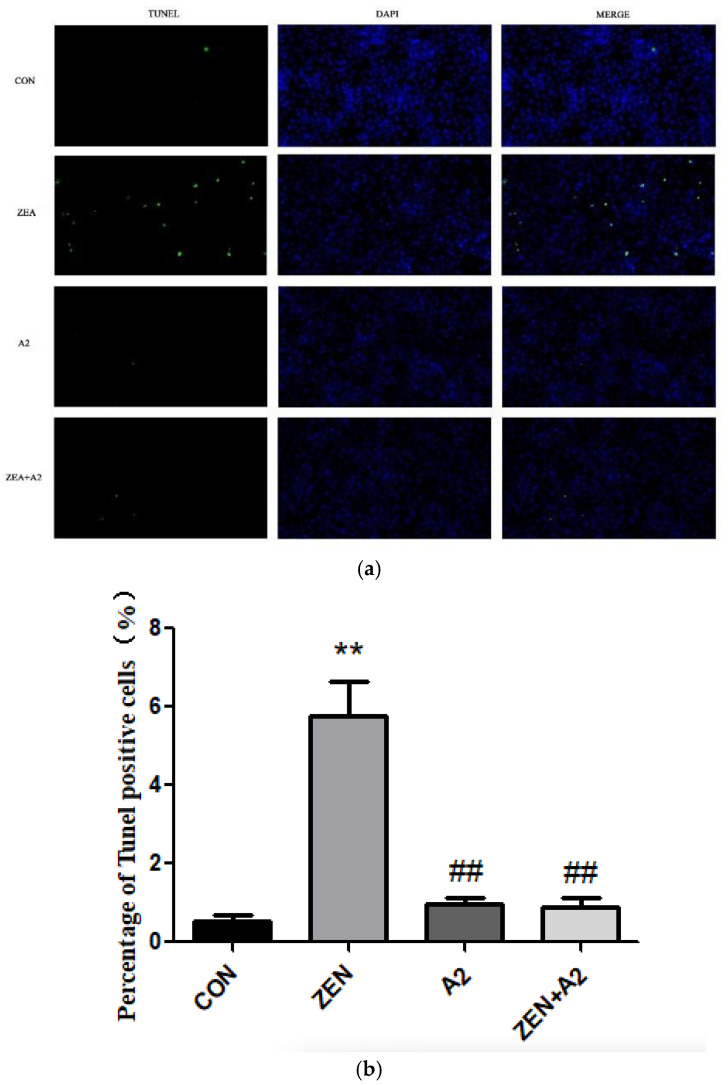
Effects of ZEA and A2 on the apoptosis of IPEC-J2 cells. Note: The images in Figure (**a**) were randomly selected under a 200× magnification field of view, Figure (**b**), detailed analysis was conducted on the proportion of TUNEL positive cells using GraphPad-prism software, and each group of experiments includes the average ± SD of at least five independent replicates (*n* = 5). Among them, “**” denotes a highly significant difference compared to the ZEA and CON groups (*p* < 0.01), while “##” denotes a highly significant difference between the A2 group or A2+ZEA group and the ZEA group (*p* < 0.01).

As shown in Figure 2, the number of TUNEL-positive cells in the ZEA group significantly increased compared to CON (*p* < 0.01), indicating that ZEA treatment significantly promoted the apoptosis of IPEC-J2 cells. Compared with the ZEA group, the number of TUNEL-positive cells in the A2 group and the ZEA+A2 group was significantly reduced (*p* < 0.01). A2 could weaken the effect of ZEA on cell apoptosis, significantly reduce the toxic damage caused by ZEA on cells, and provide an important reference for further research into the mechanism of cell apoptosis and the prevention and treatment of toxicity.

### 2.3. Detection Results of the IPEC-J2 Cell Cycle

**Figure 3:**
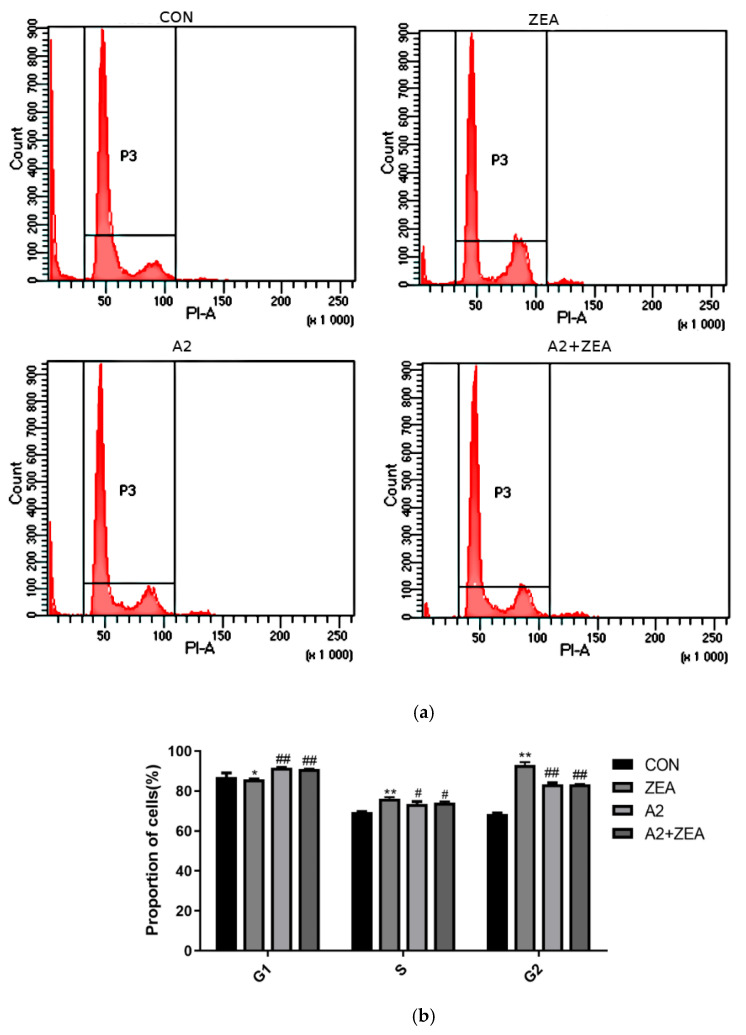
In the Figure (**a**) changes in cell cycle were detected using flow cytome. the Figure (**b**) cell cycle was analyzed using GraphPad price software.Effects of ZEA and A2 on the cell cycle of IPEC-J2 cells. Note: Flow cytometry analysis of the cell cycle distribution showed that ZEA-induced cell cycle arrest occurred in the S and G2 phases. Each group of data has an average of three independent replicates ± SD (*n* = 3). Among them, “*” indicates a significant difference compared to the ZEA and CON groups (*p* < 0.05), “**” represents a very significant difference compared to the ZEA and CON groups (*p* < 0.01), “#” denotes a significant difference between the A2 group or A2+ZEA group and the ZEA group (*p* < 0.05), and “##” represents a very significant difference between the A2 group or A2+ZEA group and the ZEA group (*p* < 0.01).

As shown in Figure 3, ZEA treatment significantly decreased the proportion of cells in the G1 phase (*p* < 0.05), while increasing the proportion of cells in the S and G2 phases (*p* < 0.01). The finding indicated that ZEA may promote cells to enter the DNA replication and division stage prematurely, thereby reducing the time for the cell growth and functional recovery. This change in the cell cycle may be related to ZEA-induced oxidative stress and cell damage. After A2 treatment, there was a significant change in the distribution of cell cycles. Compared with the ZEA group, the proportion of cells in the A2 group and A2+ZEA group significantly increased in the G1 phase (*p* < 0.01), while the proportion in the S and G2 phases was remarkably decreased (*p* < 0.05 and *p* < 0.01). The addition of A2 could correct the cell cycle abnormalities caused by ZEA, allowing cells to stay longer in the growth and functional recovery stages, thus helping to reduce the oxidative damage of ZEA to cells.

### 2.4. Results of Transcriptomics Analysis

#### 2.4.1. Repetitive Correlation Assessment

**Figure 4:**
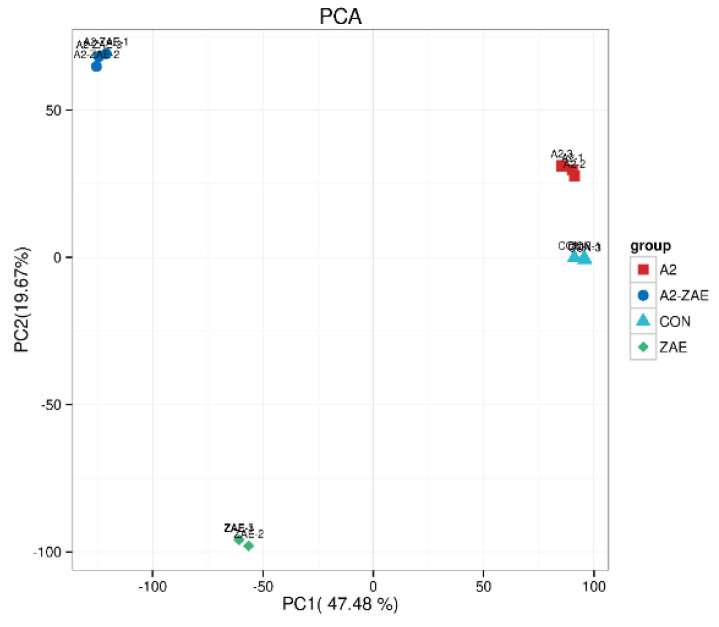
Repetitive correlation assessment in transcriptomics. Note: Each coordinate represents an independent principal component, and the corresponding percentage reflects the contribution of each principal component to the sample differences. Each data point represents a sample, while samples in different groups are distinguished by different colors and shapes.

A principal component analysis (PCA) was used to analyze transcriptomic data. As shown in Figure 4, the sample points of the CON and A2 groups are relatively close, implying that the addition of A2 had a small effect on the gene expression in IPEC−J2 cells.

#### 2.4.2. Analysis of Differential Gene Expression

**Figure 5:**
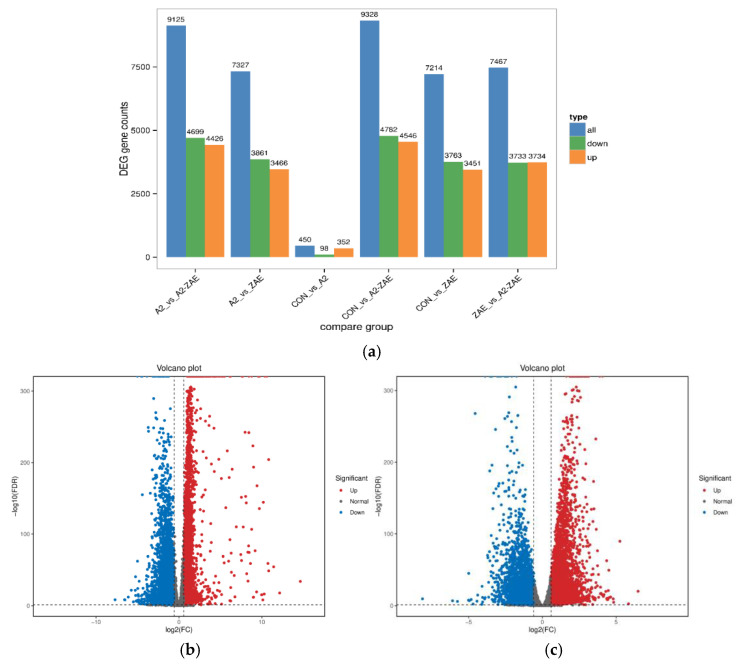
Analysis of differential gene expression. Note: The horizontal axis of Figure (**a**) represents different sets of differentially expressed genes, with blue, green, and orange distinguishing the total number of genes, the number of upregulated and downregulated genes, and the vertical axis representing the specific number of genes. In the differential expression volcano diagram of Figure (**b**,**c**), each data point corresponds to a gene. In the diagram, the horizontal axis stands for the logarithmic difference in the gene expression between the two samples, while the vertical axis denotes the negative logarithmic difference in changes of gene expression. The blue data points represent downregulated differentially expressed genes, the red represents upregulated differentially expressed genes, and the gray denotes genes without differentially expressed genes.

For the comparison between the CON group and the ZEA group (Figure 5a), a total of 7214 genes were detected showing significant differences, with 3451 genes upregulated and 3763 genes downregulated. In the comparison between the ZEA group and the A2+ZEA group, a total of 7467 significantly different genes were found, including the upregulation of 3734 genes and downregulation of 3733 genes. These data show that the treatment of ZEA and A2 can significantly affect the expression of the genome.

The results of Figure 5b,c can further validate the significant effects of ZEA and A2 treatment on genome expression. Compared to the number of unchanged genes, there are a large number of genes with significant changes in expression levels. This discovery emphasizes the profound effects of these two treatments on the cell gene expression, making it necessary for us to assess the treatment effects and potential biological mechanisms of action thereof.

#### 2.4.3. Analysis of Differential Gene Enrichment

**Figure 6:**
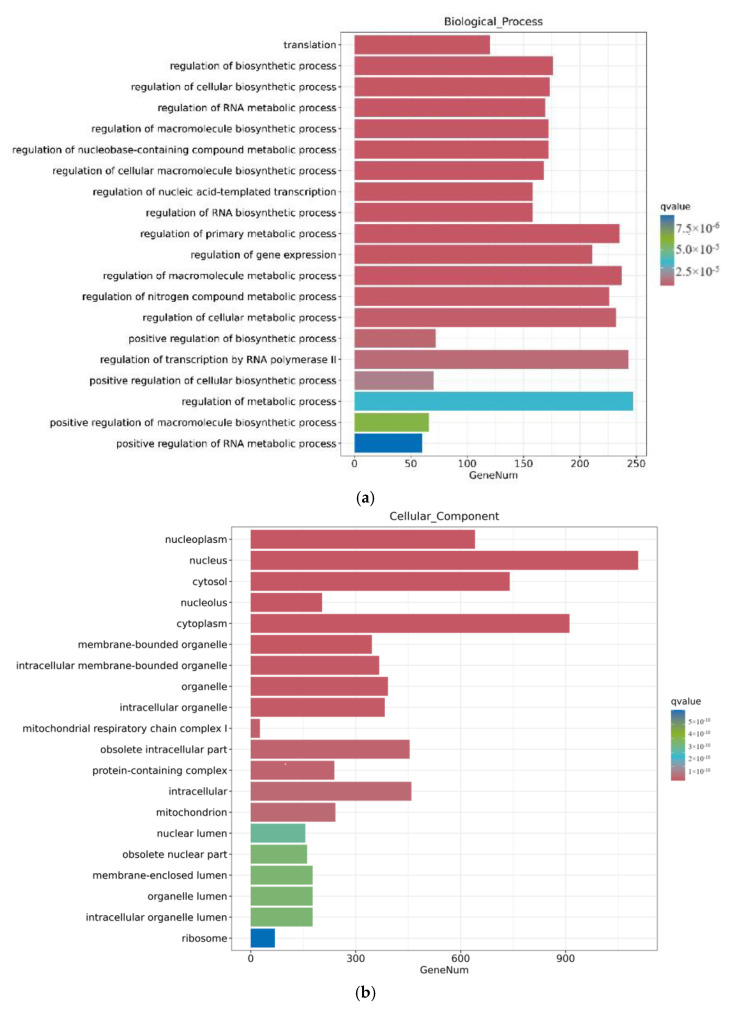

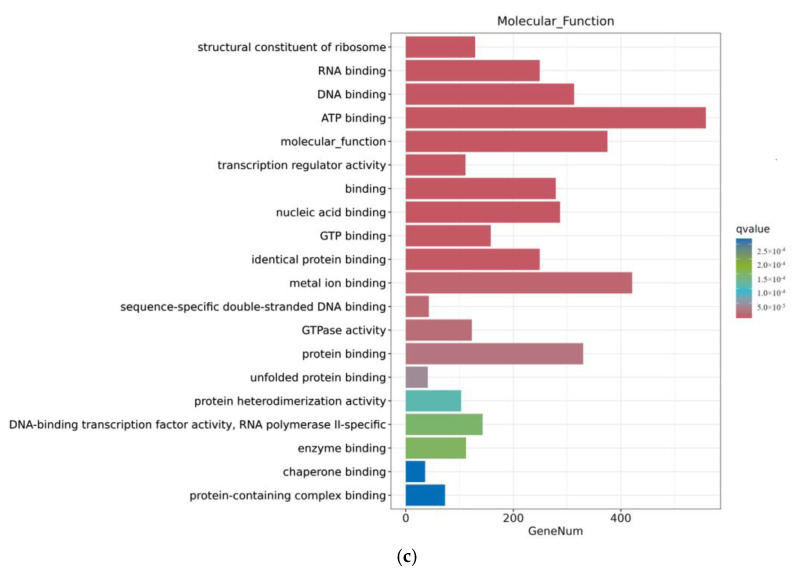
GO enrichment analysis of differential genes between the CON group and ZEA group. Note: The horizontal axis Gene Num in the figure annotates the number of genes, while the vertical axis lists each GO annotation. To better distinguish between the results, columns of different colors were used in the figure to represent the *q* value of the hypergeometric test. (**a**): Biological process, (**b**): Cellular Cpmponent, (**c**): Molecular Function.

Through a GO enrichment analysis, as shown in Figure 6, after 12 h of ZEA treatment, a series of biological processes involving gene regulation were discovered in terms of biological processes. This includes the regulation of metabolic processes, regulation of cellular metabolic processes, and regulation of macromolecular processes. These results indicate that the treatment of ZEA affects the regulation of cellular processes and regulation of macroporous processes, showing that ZEA plays an important role in cellular signaling and metabolic regulation. Secondly, in terms of cellular composition, ZEA treatment triggered significant changes in the internal composition of cells. These changes covered important cellular components such as the nucleus, cycloplast, cyclosol, and nucleoplast. This indicates that ZEA has a significant effect on the internal environment of cells and the composition of certain organelles. In terms of molecular functions, the role of ZEA involves multiple key molecular functions. This includes ATP binding, metal ion binding, and molecular functions. These findings further prove that ZEA, as a toxic substance, may interfere with important biological pathways such as neural conduction, energy metabolism, and protein synthesis in cells.

**Figure 7:**
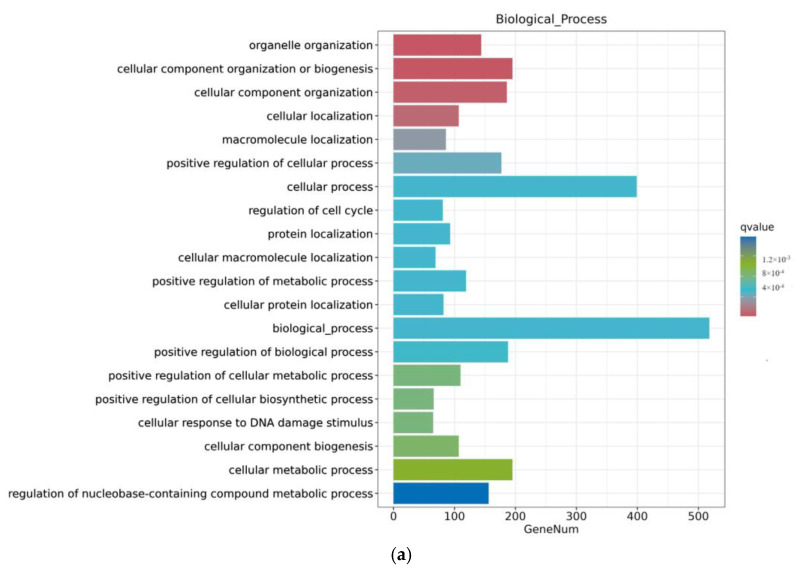

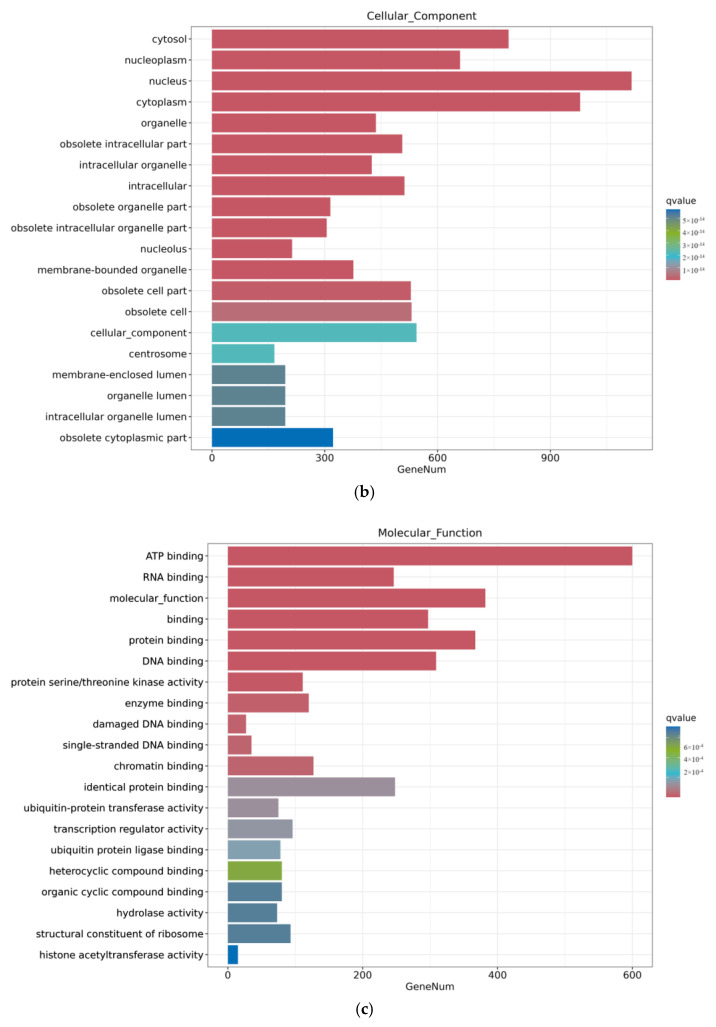
GO enrichment analysis of differential genes between the ZEA group and A2+ZEA group. Note: The horizontal axis Gene Num in the figure annotates the number of genes, while the vertical axis lists each GO annotation. To better distinguish between the results, columns of different colors were used in the figure to represent the *q* value of the hypergeometric test. (**a**): Biological process, (**b**): cellular component, (**c**): molecular function.

As shown in Figure 7, the ZEA group also experienced significant changes in gene function after A2 pretreatment for three hours. In terms of biological processes, there were more genes involved in functional changes in biological processes and cellular processes; in terms of cellular composition, the number of genes involved in the nucleus, cycloplast, cyclosol, and nucleoplast remained relatively high; in terms of molecular function, the number of metal ion binding genes significantly decreased.

#### 2.4.4. KEGG Annotation Analysis

**Figure 8:**
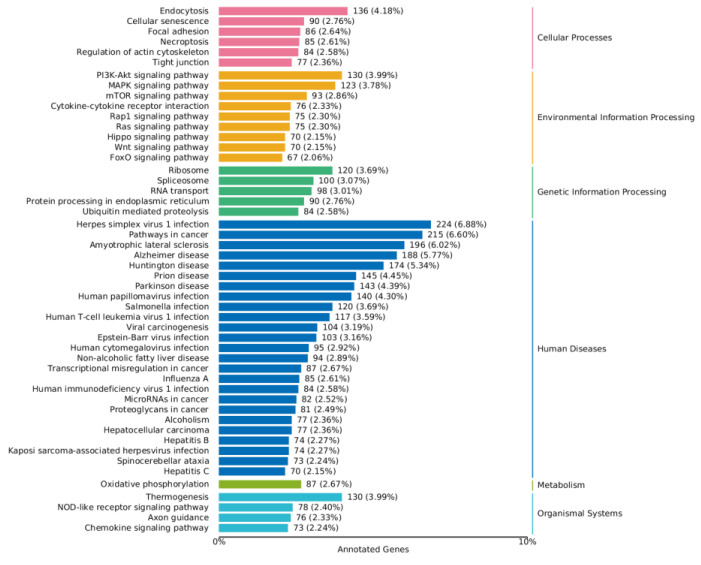
KEGG enrichment analysis of differential genes between the CON group and ZEA group. Note: The left coordinate indicates the specific name of the KEGG metabolic pathway, while the right coordinate represents the primary classification name related to the pathway. The horizontal coordinate denotes the number of genes annotated under this pathway and its proportion to the total number of annotated genes.

**Figure 9:**
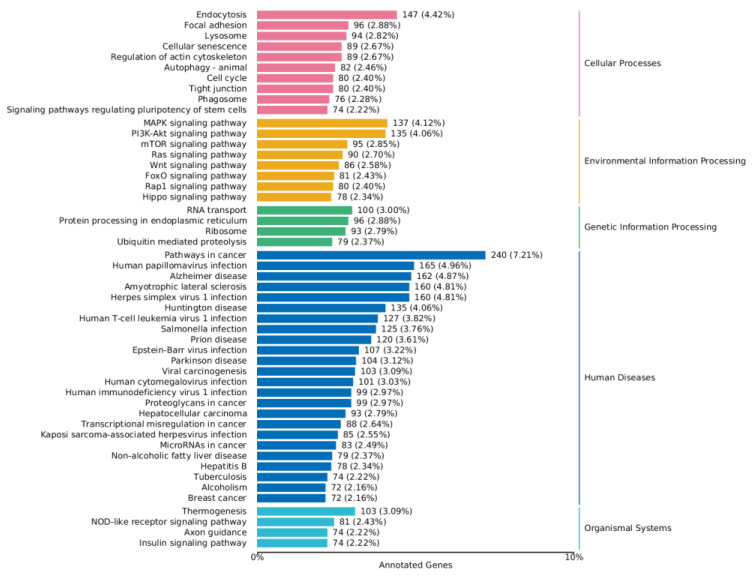
KEGG enrichment analysis of differential genes between the ZEA group and A2+ZEA group. Note: The left coordinate indicates the specific name of the KEGG metabolic pathway, while the right coordinate represents the primary classification name related to the pathway. The horizontal coordinate denotes the number of genes annotated under this pathway and its proportion to the total number of annotated genes.

As shown in Figure 8 and Figure 9, a series of KEGG pathways can be significantly enriched in the MAPK pathway, PI3K-AKT pathway, FoxO pathway, mTOR pathway, and Wnt signaling pathway. The finding indicates that ZEA may trigger inflammatory and oxidative stress responses within cells. The significant changes in these pathways may be related to the regulation of cellular immune functions, cell growth, and metabolic processes.

### 2.5. Expression of Related Genes and Proteins

**Figure 10:**
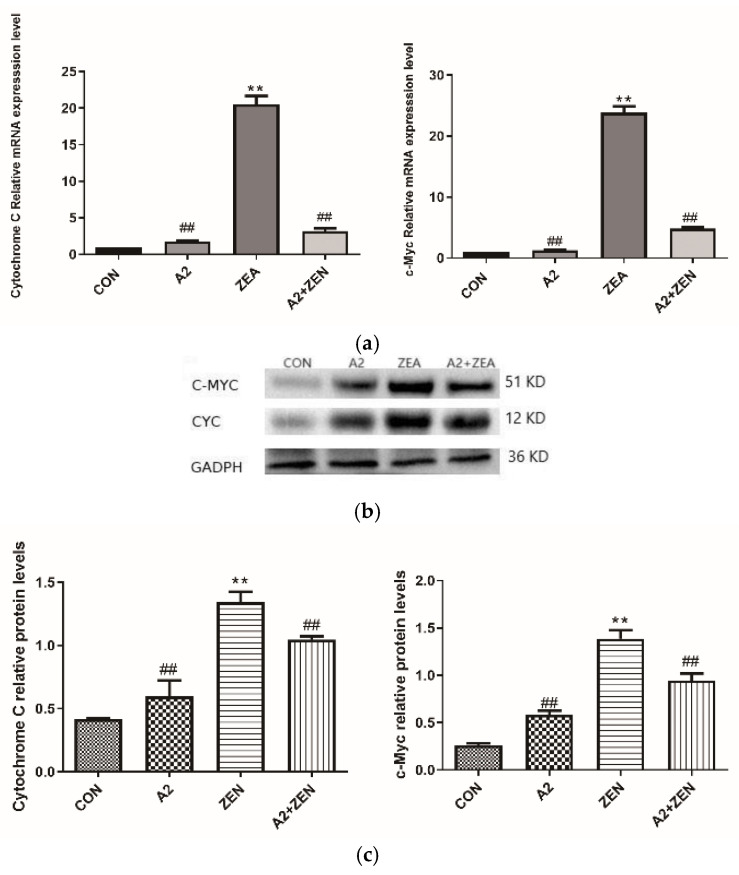
Expression of cell cycle-related genes. Note: Figure (**a**) shows the mRNA expression level of the target gene, while Figure (**b**,**c**) present the protein expression and statistical analysis of the gene, respectively. Each experiment consists of three independent replicates, with “**” indicating a significant difference compared to the ZEA and CON groups (*p* < 0.01), and “##” representing a significant difference between the A2 group or A2+ZEA group and the ZEA group (*p* < 0.01).

As shown in Figure 10a, compared to the CON group, the mRNA expression levels of c-Myc and Cytochrome C in the ZEA group significantly increased (*p* < 0.01). The results suggested that the gene expression of c-Myc and Cyto C in cells was significantly regulated when subjected to ZEA treatment. C-Myc is an important transcription factor that plays a crucial role in cell cycle regulation and cell proliferation. High expression levels of Cyto C may indicate activation of the cell apoptosis pathway, leading to an increase in cell apoptosis, which coincides with the TUNEL staining results of this experiment. These results suggest that ZEA may have affected the cell cycle and apoptosis pathways. On the contrary, the A2 and A2+ZEA groups showed a significant decrease in the mRNA expression levels of c-Myc and Cyto C compared to the ZEA group (*p* < 0.01). These results may imply that the intervention of probiotic A2 can reverse the increased expression of c-Myc and Cyto C caused by ZEA, help restore the normal cell cycle and apoptosis pathways, and alleviate the adverse effects of ZEA on cells. Figure 10b,c depict the protein expression levels of the c-Myc and Cyto C genes, which are consistent with the mRNA expression trends of the corresponding genes.

**Figure 11:**
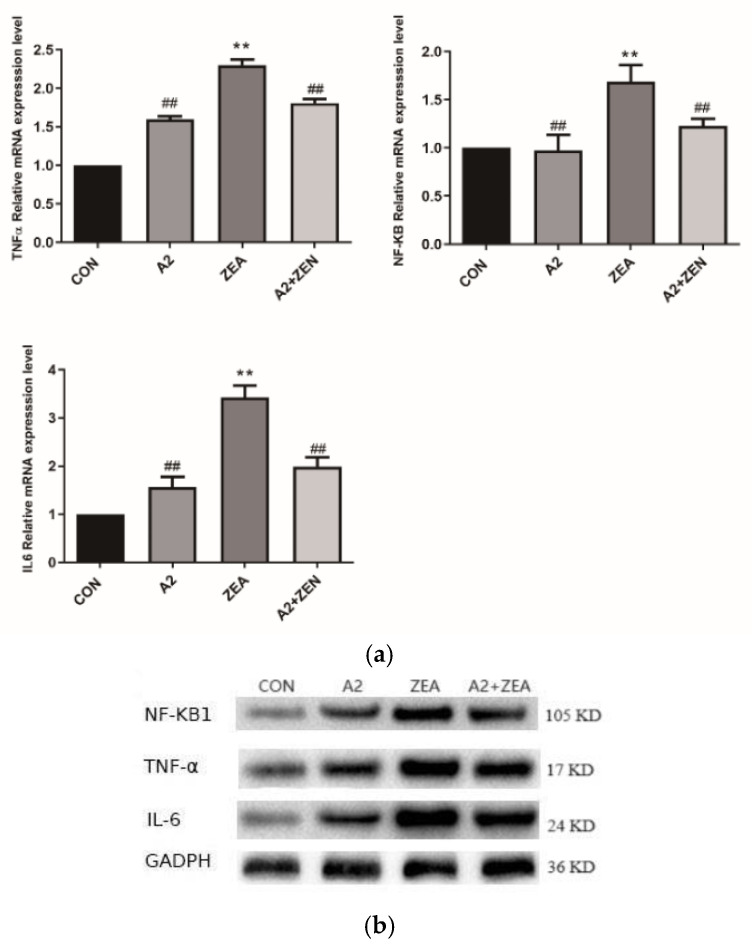

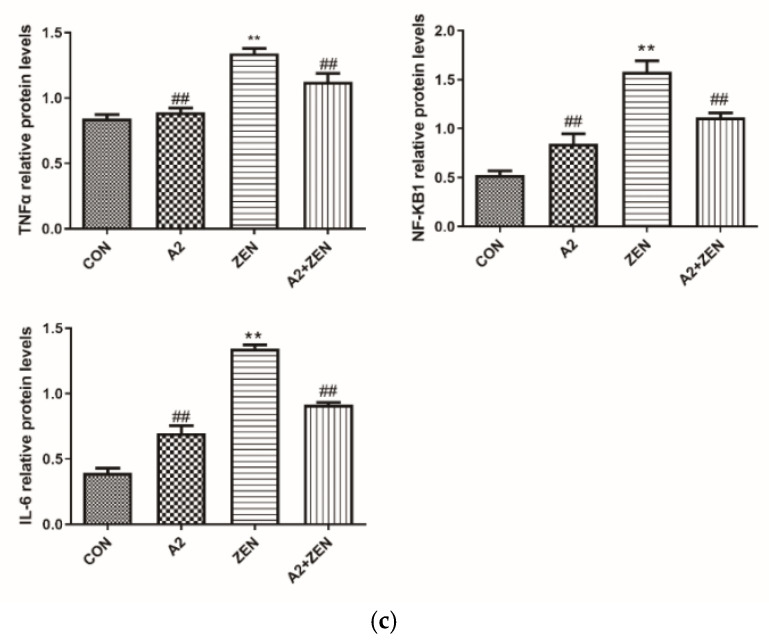
Expression of inflammatory genes. Note: Figure (**a**) shows the mRNA expression level of the target gene, while Figure (**b**,**c**) display the protein expression and statistical analysis of the gene, respectively. Each experiment consists of 3 independent replicates, “**” indicates a very significant difference compared to the ZEA and CON groups (*p* < 0.01), and “##” denotes a very significant difference between the A2 group or A2+ZEA group and the ZEA group (*p* < 0.01).

IL6 and TNF-α are key regulatory factors in the inflammatory process, and NF-κB1 is an important regulatory factor in the inflammatory signaling pathway. As illustrated in Figure 11a, compared to the CON group, the ZEA group showed significant differences in IL6 TNF-α and NF-κB1. The mRNA expression level of ZEA significantly increased (*p* < 0.01), indicating an enhanced inflammatory response in the ZEA group. On the other hand, compared to the ZEA group, the IL-6, TNF-α, and NF-κB levels of mRNA expression were significantly decreased (*p* < 0.01). This finding indicates that probiotic A2 has a positive effect on suppressing an inflammatory response. Figure 11b,c demonstrate that IL6, TNF-α, and NF-κB1 protein expression levels of genes are consistent with the mRNA expression trends of corresponding genes.

**Figure 12:**
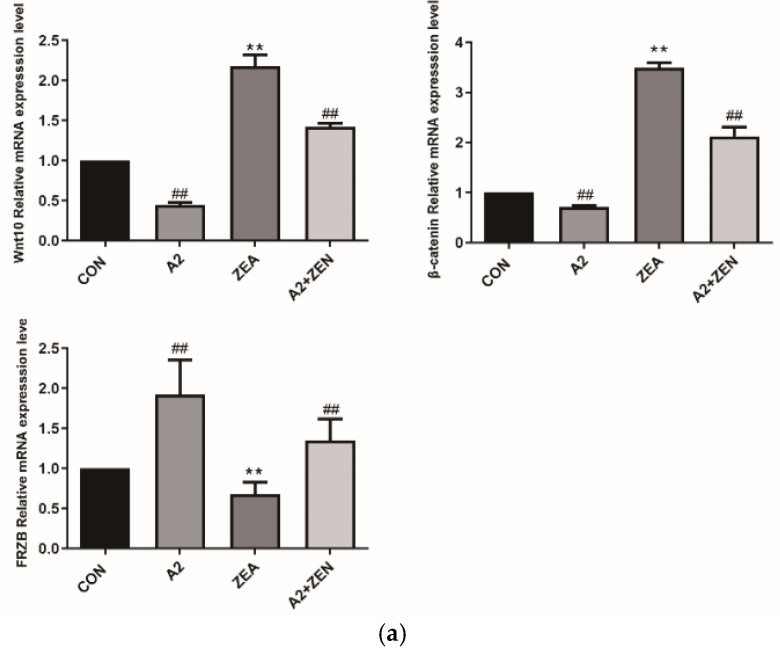

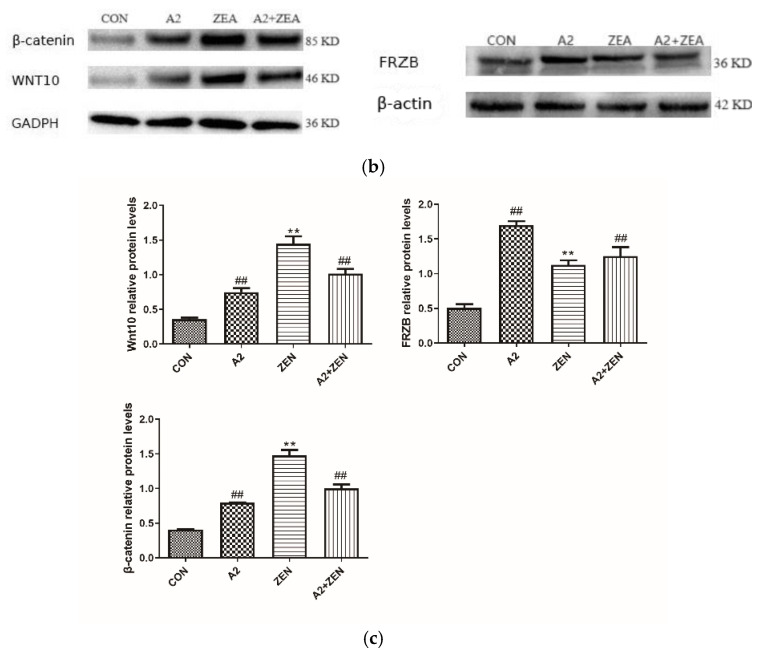
Gene expression of the Wnt signaling pathway. Note: Figure (**a**) shows the mRNA expression level of the target gene, while Figure (**b**,**c**) present the protein expression and statistical analysis of the gene, respectively. Each experiment consists of 3 independent replicates, “**” indicates a very significant difference compared to the ZEA and CON groups (*p* < 0.01), and “##”denotes a very significant difference between the A2 group or A2+ZEA group and the ZEA group (*p* < 0.01).

As shown in Figure 12a, compared with the CON group, it was found that for Wnt10 and β-catenin, the mRNA level of expression of FRZB was significantly upregulated in the ZEA group (*p* < 0.01), while the expression level of FRZB was significantly decreased (*p* < 0.01). This result indicated that under the conditions of ZEA treatment, the Wnt/FRZB/β-catenin pathway was activated. Compared with the A2+ZEA group, the level of mRNA expression of FRZB significantly decreased in the A2+ZEA group (*p* < 0.01), while the expression level of FRZB significantly increased (*p* < 0.01). This implied that the intervention of probiotic A2 affects the Wnt/FRZB/β-catenin pathway, leading to significant changes in the expression levels of key factors in the pathway. Figure 12b,c show that Wnt10 β-catenin and protein expression levels of the FRZB gene are consistent with the mRNA expression trends of the corresponding genes.

## 3. Discussion

ROS, as a type of ROS molecule within cells [24], play a crucial role in oxidative stress, and antioxidants help neutralize these free radicals [25]. Under normal physiological conditions, ROS have important signaling and regulatory functions; however, under intracellular oxidative stress, the production of ROS significantly increases, leading to intracellular oxidative stress [26]. ROS can interact with DNA, proteins, and lipids, ultimately resulting in cell apoptosis or necrosis, as well as changes in the cell cycle [27,28,29]. In response to the threat of ROS, organisms are equipped with a complex antioxidant system, including SOD, GSH-PX, T-AOC, and CAT [30]. These antioxidant substances work together to help neutralize ROS, maintain redox balance, and prevent cell damage [31,32].

Antioxidants can enhance the activity of intracellular antioxidant enzymes and maintain a redox balance by promoting the synthesis of antioxidant enzymes [33]. These substances work together to enhance the antioxidant capacity of cells, thereby reducing the toxic damage caused by fungal toxins [34]. Antioxidants include T-SOD, GSH-PX, and CAT, which can quickly clear ROS [35,36]. Our research results are consistent with this finding. After adding A2, the expression levels of T-SOD, GSH-PX, T-AOC, and CAT in IPEC-J2 cells exposed to ZEA were significantly increased (*p* < 0.01). Secondly, A2 may maintain the intracellular redox balance by decreasing the levels of enzymes such as MDA [15]. In this study, the expression level of MDA in the A2+ZEA group significantly increased compared to the ZEA group (*p* < 0.01). The synergistic effect of these mechanisms helps to improve the antioxidant capacity of cells and alleviate the toxic damage caused by fungal toxins. In addition, *Bacillus velezensis* A2 may reduce fungal toxin-induced ROS generation by regulating signaling pathways. In addition, A2 also eliminates free radicals within cells by enhancing the activity of antioxidant enzymes such as SOD and CAT, alleviating oxidative stress reactions, and decreasing oxidative damage within cells [37]. These mechanisms help maintain cell homeostasis and reduce cell damage.

In addition, A2 regulates various stages of the cell cycle by influencing cell cycle- related proteins such as Cyclin D, CDKs, and p21 [38]. This regulation may be achieved by altering the expression level (or activity) of these proteins to ensure that cells complete different stages of the cell cycle at appropriate time points. Finally, A2 may also mediate cell apoptosis pathways, which affects cell survival and death decisions by regulating apoptosis-related proteins such as the Bcl-2 family, c-Myc, Cytochrome C, etc. [39].

A2 inhibits the expression of inflammatory-related genes such as IL-6, TNF-α, and IL-1 β, alleviating the cellular inflammatory response induced by mycotoxins [40]. The excessive release of these inflammatory factors is associated with cell damage [41]. A2 may inhibit IL-6 and TNF-α. The production of A2 to alleviate inflammation usually involves NF-κB intervention and inhibition of the signaling NF-κB pathways. To reduce the synthesis and secretion of inflammatory factors [42], NF-κB is an important transcription factor involved in the regulation of inflammatory genes. When not stimulated-NF-κB located in the cytoplasm, but in fungal toxin-induced cellular inflammation, NF-κB activated and migrated to the nucleus, promoting the transcription of inflammation-related genes [43]. Therefore, A2 affects the NF-κB activation and nuclear translocation to suppress the inflammatory response.

In-depth research was conducted on the changes in the gene expression levels in cells treated with A2 using a transcriptome analysis. This research included identifying upregulated and downregulated genes. For the ZEA group and A2+ZEA group, a total of 7467 genes showed significant changes, involving pathways related to inflammation, oxidative stress, and cell cycle. Wnt/FRZB/β-catenin signal pathways were screened out to gain a deeper understanding of the effect of the A2 strain on the cellular gene regulation.

The Wnt/FRZB/β-catenin signaling pathway is an important signaling pathway involved in biological processes such as cell growth, differentiation, and tissue development. The core members of this pathway include the Wnt protein, Frizzled receptor family (such as FRZB), and β-catenin. Under normal circumstances, the binding of the Wnt protein can activate the Frizzled receptor, leading to β-catenin stability and the accumulation of catenin, thereby activating the Wnt/β-catenin signaling pathway. However, in some cases, FRZB can antagonize the effect of the Wnt protein, thus reducing the stability and activity of β-catenin. The abnormal activation or inhibition of this pathway is related to various diseases and biological processes, which are of great significance for studying cell signaling and physiological regulation.

After A2 intervention, the mRNA expression level of Wnt10 and β-catenin significantly decreased in the A2+ZEA group (*p* < 0.01), while the expression level of FRZB significantly increased (*p* < 0.01). This indicates that the treatment of A2 may affect Wnt/FRZB/β-catenin activity. The activity of this pathway exerts a significant influence, leading to significant changes in the expression levels of key factors in the pathway. In this experiment, the expression of Wnt/FRZB/β-catenin signaling pathways in various groups was analyzed, providing important information about trends in the expression of these genes under different processing conditions. Compared to the CON group, the mRNA expression levels of IL-6 TNF- α, IL-1β, and NF-κB were significantly increased (*p* < 0.01), which is consistent with the results of this experiment. In addition, after A2 intervention, the mRNA expression level of FRZB significantly decreased in the A2+ZEA group (*p* < 0.01), while the expression level of FRZB significantly increased (*p* < 0.01). This indicates that the treatment of A2 may affect the Wnt/FRZB/β-catenin pathway that has a significant effect, leading to significant changes in the expression levels of key factors in this pathway.

## 4. Conclusions

ZEA increases the levels of oxidative active substances ROS and MOD in IPEC-J2 cells; decreases the levels of antioxidant enzyme active substances T-SOD, GSH-PX, T-AOC, and CAT; and disrupts the intracellular redox balance. The addition of *Bacillus velezensis* A2 can eliminate oxidative active substances in IPEC-J2 cells, increase the level of antioxidant enzyme active substances in cells, and thereby maintain the intracellular redox state; it also reduces the proportion of cells suffering apoptosis and restores normal cell cycles.

*Bacillus velezensis* A2 regulates the Wnt/FRZB/β-catenin oxidation signaling pathway within IPEC-J2 cells, and significantly downregulates the level of expression of the Wnt, β-catenin gene, upregulating the level of expression of the FRZB gene. It downregulates the level of expression of inflammation-related genes IL6, TNF-α, and NF-κB1, and downregulates the levels of expression of cell transcription factor c-Myc and the cell cycle regulatory protein cytochrome C gene. These results indicate that *Bacillus velezensis* A2 reduces the oxidative and inflammatory damage to cells caused by ZEA, alleviating the adverse effects of ZEA on cell apoptosis and cell cycles; *Bacillus velezensis* A2 has played a positive role in antagonizing the damage caused by ZEA to mouse cecum and IPEC-J2 cells.

## 5. Materials and Methods

### 5.1. Test Grouping

Four groups, a blank control group (CON), ZEA toxin group (ZEA), A2 group (Bacillus velezensis A2), and detoxification group (A2+ZEA) were established. Based on previous experiments conducted by our research group, 110 μmol/L ZEA (Pribolab, Qingdao, China) was selected for this experiment and 10^11^ CFU/L A2 could be used as the experimental dosage. Among them, cells in the CON group were cultured normally for 15 h; the A2 group was first cultured with A2 strain for three hours, washed, and then cultured normally for 12 h; after three hours of normal culture of the cells in the ZEA group, ZEA was treated for 12 h; the A2+ZEA group was first cultured with A2 strain for three hours, followed by washing, and adding ZEA-treated cells for 12 h.

### 5.2. Cultivation of Test Strains and Cells

*Bacillus velezensis* A2 (A2) was isolated and stored by the Laboratory of the School of Animal Husbandry and Veterinary Medicine, Shenyang Agricultural University, China. Small intestinal porcine epithelial cells (IPEC-J2) were purchased from Shanghai Mingjin Biological Cells Co., Ltd. (Shanghai, China).

A2 was introduced into a 5 mL LB (Solarbio, Beijing, China) liquid medium using a sterile inoculation method. The cultivation conditions were 37 °C and 150 rpm, after continuous cultivation for 24 h, we inoculated A2 of 1% into 20 mL LB medium.

IPEC-J2 cells were cultured in T25 cell bottles. The culture medium contained: 89% DMEM high glucose medium (Thermo Fisher Scientific, Shanghai, China), 10% fetal bovine serum (Servicebio, Wuhan, China), 1% penicillin and streptomycin (Solarbio, Beijing, China). The samples were cultivated at 37 °C in an atmosphere containing 5% CO_2_ (*v*/*v*).

### 5.3. Detection of IPEC-J2 Cell Oxidation Indicators

During the experiment, IPEC-J2 cell oxidation indicators were detected according to the instructions supplied with the ELISA reagent kit, including ROS (Beijing Solebao Technology Co., Ltd., Beijing, China), T-SOD (Beijing Solebao Technology Co., Ltd.), GSH-PX (Shanghai Zeye Biotechnology Co., Ltd., Shanghai, China), T-AOC (Shanghai Yaji Biotechnology Co., Ltd., Shanghai, China), MDA (Beijing Solebao Technology Co., Ltd.), and CAT (Shanghai Silk Biotechnology Co., Ltd., Shanghai, China).

### 5.4. The TUNEL Method for Detecting Cell Apoptosis

We fixed cells with 4% paraformaldehyde (Solarbio, Beijing, China) at room temperature for 30 min, after washing with PBS (Solarbio, Beijing, China), then added 0.1% Triton X-100 (Solarbio, Beijing, China) to the treated cells for 10 min. A reaction mixture was prepared according to the instructions supplied with the one-step TUNEL In-Situ Apoptosis kit (Pricella, Wuhan, China), and the cell nucleus was stained. Then, the sample was observed using a fluorescence microscope (Thermo Fisher Scientist, Shanghai, China) at a green wavelength of 490 nm and apoptotic events were labeled. Image J 2.0 analysis software was used to select five fields of view at random from the cell slides of each treatment group (200×). We then performed cell counting and analyzed the proportion of TUNEL-positive cells using GraphPad-prism 8.0 software.

### 5.5. Flow Cytometry Detection of the Cell Cycle

Each experimental group was established with three independent replicates. Cells were digested with 0.25% trypsin (Yeasen, Shanghai, China), precooled with 75% ethanol (Solarbio, Beijing, China), and fixed at 4 °C for 4 h, whereafter we added a PI staining solution (50 ug/mL, green, Solarbio, Beijing, China), then incubated the samples at 4 °C in the dark for 30 min. Using an Attune CytPix imaging flow cytometer (Thermo Fisher Scientist, Shanghai, China), detection was undertaken using standard procedures; the results were analyzed using cell cycle fitting software ModFit 3.0.

### 5.6. Transcriptome Sequencing

Transcriptome sequencing consisted of four groups, each with three biological replicates. The animal total RNA was extracted according to the instruction manual supplied with the TRlzol reagent kit (Life Technologies, Framingham, MA, USA). RNA concentration and purity were measured using NanoDrop 2000 (Thermo Fisher Scientific, Wilmington, DE, USA). RNA integrity was assessed using the RNA Nano 6000 Assay Kit of the Agilent Bioanalyzer 2100 system (Agilent Technologies, Santa Clara, CA, USA).

Sequencing libraries were generated using the Hieff NGS Ultima Dual-mode mRNA Library Prep kit for Illumina (Yeasen Biotechnology (Shanghai) Co., Ltd.) following the manufacturer’s recommendations, and index codes were added to attribute sequences to each sample. The library fragments were purified with the AMPure XP system (Beckman Coulter, Beverly, MA, USA). The PCR was then performed with Phusion High-Fidelity DNA polymerase, Universal PCR primers, and Index (X) Prime. The PCR products were purified (AMPure XP system), and library quality was assessed on the Agilent Bioanalyzer 2100 system. The libraries were sequenced on an Illumina NovaSeq platform to generate 150 bp paired-end reads, according to the manufacturer’s instructions. The raw reads were further processed with a bioinformatic pipeline tool, the BMKCloud (www.biocloud.net (accessed on 11 December 2023)) online platform. A gene ontology (GO) enrichment analysis of the differentially expressed genes was implemented by the clusterProfiler packages based on a Wallenius non-central hyper-geometric distribution.

The KOBAS database and clusterProfiler 4.0 software were employed to assess the statistical enrichment of differentially expressed genes in KEGG pathways.

### 5.7. Detection of mRNA Expression Levels in IPEC-J2 Cells

The following reaction procedure was conducted following the quantitative real-time PCR reagent kit instruction manual (Takara, Shanghai, China): pre-denaturation was performed at 95 °C for 10 s, with a subsequent cyclic reaction as follows: the primer was annealed at 60 °C for 30 s, then extended at 72 °C for 15 s over a total of 40 cycles. The upstream and downstream primers of all genes in the experiment were synthesized by Sangon Biotech Company (Shanghai, China), Table 1. We calculated the mRNA expression using the ΔΔCt method and analyzed the data using Graphpad Prism8.0 software.

### 5.8. Western Blot Detection of Protein Expression

Using the Total Protein Extraction kit (Takara, Shanghai, China) to extract proteins, protein concentrations were measured using a BCA Protein Assay kit (Takara, Shanghai, China). Electrophoresis (Bio-Rad Gel Doc XR^+^ System, Bio-Rad, Hercules, CA, USA) was conducted under the following conditions: 120 V, 90 min; electric conversion (Bio-Rad Gel Doc XR^+^ System, Bio-Rad, Hercules, CA, USA) 300 mA, 90 min. The ECL luminescent liquid (Bio-Rad, Hercules, CA, USA) was adopted to perform protein imaging using a protein-imaging instrument (Bio-Rad, Hercules, CA, USA). The samples were analyzed using Photoshop and Graphpad Prism8.0 software. The names, brands, dilution ratios, species of origin, and the antibodies used in this experiment are listed in Table 2.

### 5.9. Data Statistics

The data in this experiment were all obtained from at least three independent replicates, and the data results were expressed in the form of mean ± standard deviation (X ± SEM). Statistical analysis software (SPSS20.0) was adopted to conduct a correlation analysis on the results, and the differences between the groups were compared using a one-way ANOVA. When *p* < 0.05, it proved that these data had statistical significance. In addition, the columnar statistical charts in this experiment were all plotted using the plotting software Graphpad Prism8.0.

## Figures and Tables

**Table 1:** Primer sequences.

Gene Name	FORWARD	REVERS	Size (bp)
*β-catenin*	AGCTGCTGTTCTGTTCCGTATGTC	CATGGCATTGGCTCGGTCCTG	105
*FRZB*	ACTTCCAACACGAGCCTATTAAGC	GTGGCGGTACTTGATGAGAATGG	89
*WNT10*	GCTCCTCTTCTTCCTACTGCTG	AGTCGGAGGCCCAAAATATCG	75
*β-actin*	CTGTCCCTGTATGCCTCTG	TTGATGTCACGCACGATT	221

**Table 2:** Specific situation of antibodies.

Antibody Name	Brand	Dilution Ratio	Species
β-catenin	Abclonal (Wuhan, China)	1:1000	Mouse
Wnt10	Abclonal (Wuhan, China)	1:1000	Mouse
FRZB	Abclonal (Wuhan, China)	1:6000	Rabbit
GADPH	Abclonal (Wuhan, China)	1:1000	Mouse
β-actin	Abclonal (Wuhan, China)	1:1000	Mouse
Anti mouse IgG	Sangon Biotech (Shanghai, China)	1:10,000	Mouse
Anti rabbit IgG	Sangon Biotech (Shanghai, China)	1:10,000	Rabbit

## Data Availability

Data are contained within the article.
